# Structural biology workflow for the expression and characterization of functional human sodium glucose transporter type 1 in *Pichia pastoris*

**DOI:** 10.1038/s41598-018-37445-2

**Published:** 2019-02-04

**Authors:** Albert Suades, Antonio Alcaraz, Esteban Cruz, Elena Álvarez-Marimon, Julian P. Whitelegge, Joan Manyosa, Josep Cladera, Alex Perálvarez-Marín

**Affiliations:** 1 Biophysics Unit, Department of Biochemistry and Molecular Biology, School of Medicine, Universitat Autònoma de Barcelona, 08193 Cerdanyola del Vallés, Catalonia Spain; 2 Laboratory of Molecular Biophysics, Department of Physics, Universitat Jaume I, 12071 Castellón, Spain; 3 The Pasarow Mass Spectrometry Laboratory, The NPI-Semel Institute, David Geffen School of Medicine, UCLA, 760 Westwood Plaza, Los Angeles, CA 90095 USA; 4 Present Address: Department of Biochemistry and Biophysics, Stockholm University, SE-10691 Stockholm, Sweden

## Abstract

Heterologous expression of human membrane proteins is a challenge in structural biology towards drug discovery. Here we report a complete expression and purification process of a functional human sodium/D-glucose co-transporter 1 (hSGLT1) in *Pichia pastoris* as representative example of a useful strategy for any human membrane protein. hSGLT1 gene was cloned in two different plasmids to develop parallel strategies: one which includes green fluorescent protein fusion for screening optimal conditions, and another for large scale protein production for structural biology and biophysics studies. Our strategy yields at least 1 mg of monodisperse purified recombinant hSGLT1 per liter of culture, which can be characterized by circular dichroism and infrared spectroscopy as an alpha-helical fold protein. This purified hSGLT1 transports co-substrates (Na^+^ and glucose) and it is inhibited by phlorizin in electrophysiological experiments performed in planar lipid membranes.

## Introduction

Membrane proteins play key roles in a wide range of cellular processes in eukaryotic and prokaryotic cells, accounting for 20–30% of the genome coding proteins^1,2^. Human membrane proteins are obviously relevant as biomarkers for diseases caused by protein malfunctioning and as the principal targets for pharmacological intervention^3,4^. Knowledge on the tridimensional structure of membrane proteins aids the design of drugs to open therapeutic intervention windows but despite the efforts and several structural biology breakthroughs, few structures of human membrane proteins have been resolved^5,6^. Challenging as it is, the structural biology of membrane proteins poses a major challenge, which is the expression and isolation of functional human membrane proteins. Choice of recombinant expression host can be crucial for structure determination of human membrane proteins^7^. Human membrane proteins are poorly expressed heterologously in prokaryote systems, facing obstacles such as post-translational modifications^8^. Thus, eukaryotic systems are better suited for human membrane protein expression, and so far the most successful system-of-choice has been insect cells^7^. Yeast has been the second-best heterologous expression system, and within yeast, *Pichia pastoris* has been the most successful^7,9^. *P. pastoris* has several advantages compared to insect cells for expression of large membrane protein amounts, especially regarding lab handling, molecular biology, and instrumentation requirement^10,11^. All these advantages allow better screening of expression and isolation conditions. Other successful structural biology screening strategies have been developed to optimize the purification process towards the final goal of tridimensional structure resolution, such as the use of green fluorescent protein (GFP) quicker screening of expression and solubilization conditions^12–14^. Integration of several experimental approximations is key to define successful strategies for the structural biology of human membrane proteins.

**Figure 1:**
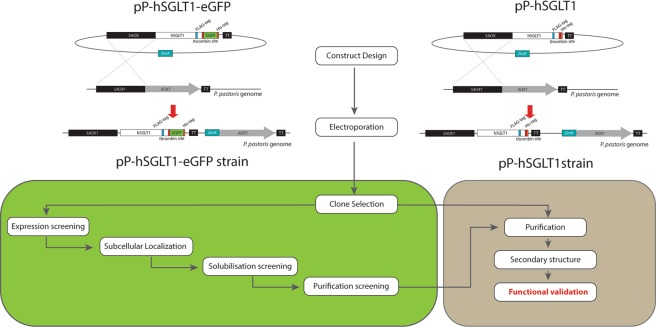
Overview of the hSGLT1 expression and characterization strategy. Notice the parallel strategy using the pP-hSGLT1-eGFP and pP-hSGLT1 vectors.

Human sodium glucose transporter 1 (hSGLT1) belongs to the solute sodium symporters (SSS) subfamily within Amino acid-Polyamine-organoCation (APC) superfamily of transporters. hSGLT1 is a member of the SLC5 gene family and was the first member to be cloned^15^. This transporter has been widely studied and related to diseases, such as Glucose-Galactose Malabsorption (GGM) or diabetes^16,17^. The structure for hSGLT1 could not be solved because poor expression levels were achieved, but the prokaryote orthologue from *Vibrio parahaemolyticus* (vSGLT) has been crystallized and the structure resolved^18^. Although the prokaryotic vSGLT structure is a relevant model, advances in the structure determination for hSGLT1 are key for human therapy and pharmacology drug design purposes. Here we report a full screening strategy (from expression host for protein source to functional protein validation) towards biophysical and structural biology studies for the expression of hSGLT1 which may be useful for any other membrane electrogenic transport proteins (Fig. 1).

## Results

### Selection of multiple copy recombinant genes of *P. pastoris* transformants

**Figure 2:**
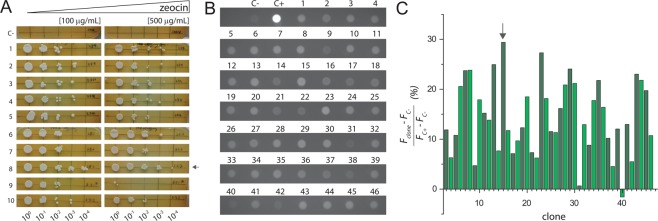
Clone selection. (**A**) Drop test in YPD plates with 100 µg/mL and 500 µg/mL of zeocin after 2–3 days of growth at 30 °C. On the left, plates with 100 µg/mL zeocin and, on the right, plates with 500 µg/mL zeocin. Each plate presented a non-transformed SMD1168H serial dilution as negative control (C−). Each number represents a tested clone and (C−) a non-transformed SMD1168H colony. A dilution factor of 10x was done for each lane, starting from left to right. (**B**) MM plates after 48 hours at 30 °C. Each dot represents a different tested clone from pP-hSGLT1-eGFP transformation except the controls (C−) and (C+). Negative control (C−) is a non-transformed SMD1168H *P. pastoris* colony. Positive control (C+) is a transformed colony of pP-eGFP empty vector. (**C**) Densitometry values of tested colonies are expressed in relative fluorescence units (RFU).

Linearized vector containing hSGLT1 was electroporated in SMD1168H to promote integration in the *AOX* locus of *P. pastoris* genome (Fig. 1) allowing for positive insertion events using zeocin. Clone screening using a serial dilution at low (100 µg/mL) and high (500 µg/mL) zeocin concentration allows the selection of multiple integration events due to variable number of copies of the bleomycin gene, which drives zeocin resistance. For pP-hSGLT1, a serial dilution in YPD medium with zeocin was done (Fig. 2A). Non-transformed SMD1168H as negative control did not grow while the rest of tested transformed clones did grow.

All tested clones grew in presence of 100 µg/mL of zeocin, but Clone 8 grew in the last dilution (10^−4^). Clone 8 fitness was confirmed at 500 µg/mL of zeocin and, therefore, the C8 strain was selected for protein expression. For pP-hSGLT-eGFP, in plate protein expression induction method was done and we were able to select clones using the fluorescence due to GFP expression (Fig. 2B). The negative control (non-transformed SMD1168H) observed in the plate reflects the intrinsic fluorescence of cells (background noise) while the positive control (pP-eGFP empty vector) expressing soluble eGFP (brightest in Fig. 2B) allows to set a relative fluorescence maximum for quantification (Fig. 2C), which reveals Clone 15 as the most intense amongst the ones transformed with pP-hSGLT-eGFP.

### Subcellular protein localization

**Figure 3:**
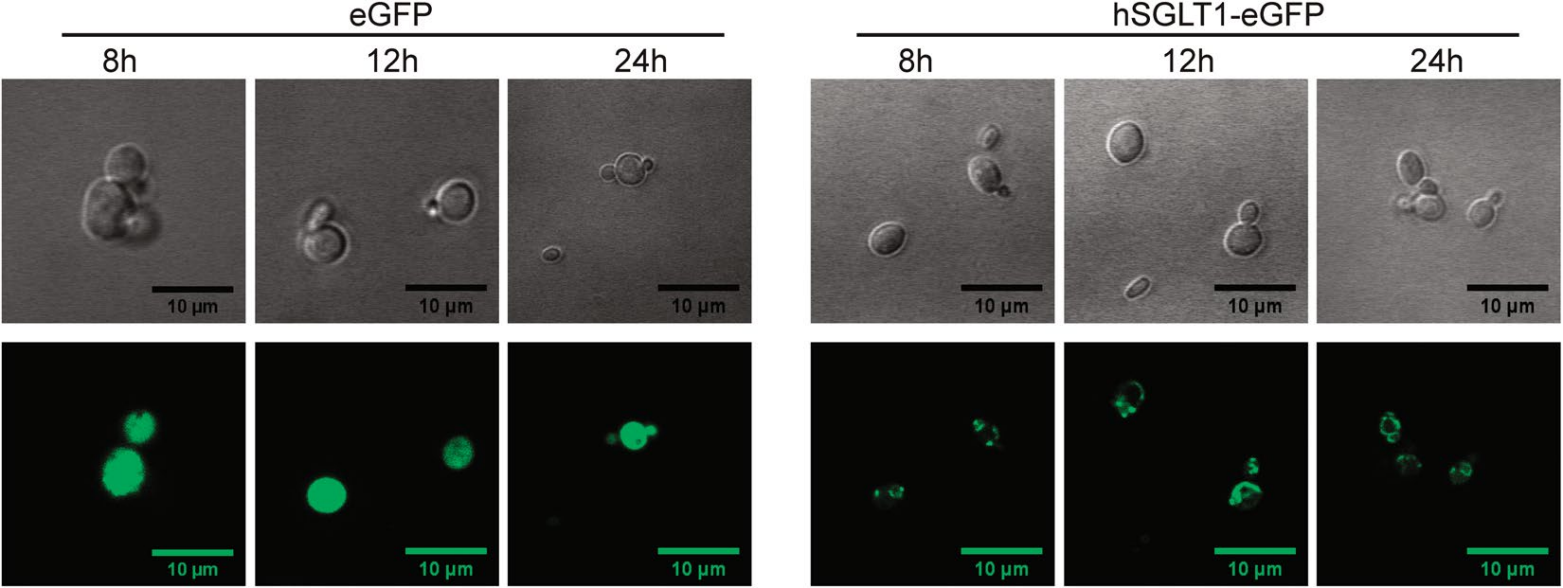
GFP fluorescence for subcellular localization. Representative fields of pP-eGFP (eGFP) and pP-hSGLT1-eGFP (hSGLT1-eGFP) strains after methanol induction in MM media at: 8, 12 and 24 hours from left to right. A bright field image is on the top and each corresponding confocal image is represented on the bottom.

Protein expression of pP-hSGLT-eGFP was analyzed by confocal microscope and compared to GFP expression (Fig. 3). GFP expression shows a diffuse pattern of expression typical for a soluble protein while hSGLT1-eGFP displays a fluorescence that is more localized in dots or patches, corresponding to the expression of proteins in membrane compartments.

### Detergent screening

**Figure 4:**
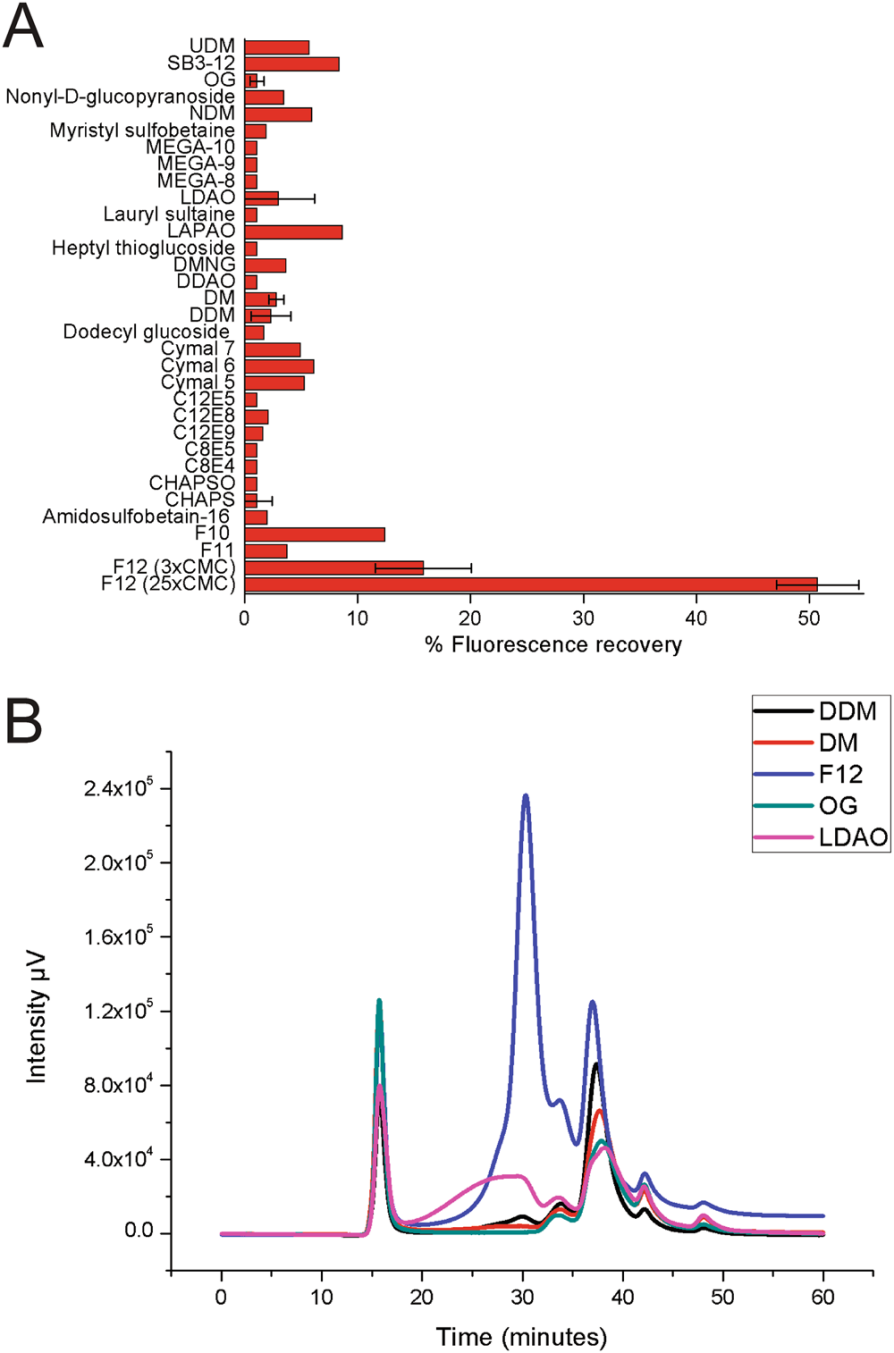
Detergent screening. (**A**) Percentage of fluorescence of solubilized membranes compared to lysate total fluorescence after solubilization with different detergents. Samples were read in a plate reader with a combination of excitation and emission filters of 485/520 nm, respectively. UDM (n-Undecyl-β-D-Maltopyranoside), SB3-12 (Sulfobetaine 3-12), OG (Octyl-β-Glucoside), NDM (n-Nonyl-β-D-Maltopyranoside), MEGA 8, 9, 10 (N-Octanoyl, N-nonanoyl, N-decadyol)-(N-Methylglucamine), LDAO (N,N-Dimethyldodecylamine N-Oxide), LAPAO (3-Dodecylamido-N, N′-Dimethylpropyl Amine Oxide), DMNG (Decyl Maltose Neopentyl Glycol), DDAO (Decyldimethylamine-N-Oxide), DM (Decyl-β-D-Maltopyranoside), DDM (Dodecyl-β-D-Maltoside), Cymal 7 (7-Cyclohexyl-1-Heptyl-β-D-Maltoside), Cymal 6 (6-Cyclohexyl-1-Hexyl-β-D-Maltoside), Cymal 5 (5-Cyclohexyl-1-Pentyl-β-D-Maltoside), C12E5 (Dodecyl Pentaethylene Glycol Ether), C12E8 (Octaethylene Glycol Monododecyl Ether), C12E9 (Dodecyl Nonaethylene Glycol Ether), C8E5 (Tetraethylene Glycol Monooctyl Ether), C8E5 (Octyl Pentaethylene Glycol Ether), CHAPS (3-[(3-Cholamidopropyl) Dimethylammonio]-1-Propanesulfonate), CHAPSO (3-[(3-Cholamidopropyl) Dimethylammonio]-2-Hydroxy-1-Propanesulfonate), F10 (n-Decylphosphocholine), F11 (n-Undecylphosphocholine), F12 (n-Dodecylphosphocholine). (**B)** FSEC chromatogram in a Superdex 200 HR column of membranes solubilized for 2 hours with selected detergents at 1% (w/v) concentration.

Membranes of pP-hSGLT1-eGFP solubilized in different detergents (Fig. 4A) were subjected to FSEC in a Superose 6 gel filtration column (Fig. 4B). The FSEC profiles for representative detergents are shown in Fig. 4B. Most of the detergents show a chromatogram with free eGFP as the predominant form (>35 minutes elution time), except for Fos-12, which solubilized hSGLT1-eGFP the most (Fig. 4A) and shows a predominant peak at 30 min. elution time in FSEC profile (Fig. 4B), corresponding to hGSLT1-eGFP. Accordingly, Fos-12 was the detergent-of-choice for protein solubilization.

### Protein purification

**Figure 5:**
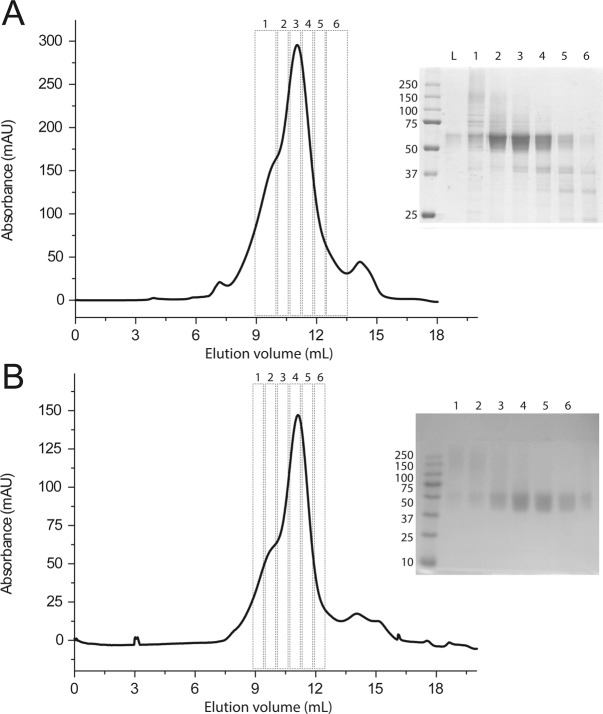
hSGLT1 purification. (**A**) SEC chromatography after Ni-NTA purification of WT-hSGLT1 with a Superdex 200 HR column of 25 mL and its corresponding SDS-PAGE on the right. Protein was run in TKCL at 0.2% (w/v) of Fos-12 at a flow of 0.4 mL/min. SEC collected fractions of 500 µL (2–5) and 1 mL (1 and 6) are represented on the SDS-PAGE. (**B)** SEC chromatography after Ni-NTA and FLAG-tag purification of WT-hSGLT1 with a Superdex 200 HR column of 25 mL and its corresponding SDS-PAGE on the right. Protein was run in TKCL at 0.2% (w/v) of Fos-12 at a flow of 0.4 mL/min. SEC collected fractions of 500 µL (1–6) were loaded and resolved in a SDS-PAGE.

Taking advantage of the eGFP fluorescence, we used the pP-hSGLT1-eGFP strain for optimization of protein expression, detergent screening and subcellular protein localization. The obvious follow-up strategy is to use the pP-hSGLT-eGFP strain to scale-up the protein purification process and remove the eGFP and 8XHis tags in subsequent steps by using proteases (Fig. 1). However, for this specific strain the thrombin digestion to remove the tags is not efficient enough (data not shown). At the point when all screenings have been made, it is important to have a contingency plan to proceed with the hSGLT1 protein purification. Thus, for large-scale protein purification we used the pP-hSGLT strain using the conditions for expression, and solubilization screened with the pP-hSGLT1-eGFP strain. Enrichment of hSGLT1 from Ni-NTA IMAC purification (Fig. S1A) eluted at 150 mM imidazole were pooled, concentrated and injected into a Superdex S200 gel filtration column (Fig. 5A). A homogenous single peak of protein is appreciated in the SEC centered at 11 mL and with a small shoulder at 9.5 mL. Fractions loaded in a SDS-PAGE are represented in Fig. 5A. The material loaded in the gel filtration (L) showed a single diffused band with an apparent molecular weight of 55 kDa (main protein-band in the Ni-NTA). To discard the effect of potential protein glycosylation, we mutated Asn248 to Ala and no remarkable effects were observed in SEC nor SDS-PAGE (Fig. S1B). In the first elution fraction (1, Fig. 5A), several bands are appreciated at: 150 kDa, 80 kDa, 75 kDa and 55 kDa. In subsequent fractions (2 and 3, Fig. 5A), the higher molecular weight band at 150 kDa vanishes while other bands (80 and 75 kDa) decay in intensity (4, Fig. 5A) but the diffused band at 55 kDa increases in intensity. A lower molecular weight band is also observed at 37 kDa. Those two bands increase in intensity in later fractions (4 and 5, Fig. 5A) while the main diffused band at 55 kDa starts to fade. In F3, it can be appreciated that two thin and low intensity bands are observed at 50 kDa which were mixed with this main diffused band at 55 kDa. Rough molecular weight estimation using gel filtration standards (Fig. S1C) indicates that the 9.5 and 11 mL fractions in Fig. 5A correspond to large protein-detergent complexes at 376 and 204 kDa, respectively (Fig. S1D). Taking advantage of the FLAG tag engineered within the ORF of hSGLT1 (Fig. 1), we performed a polishing step using a FLAG-Tag resin after Ni-NTA IMAC and prior to Superdex S200 gel filtration (Fig. 5B), in order to remove the impurities observed in Fig. 5A. The chromatogram in Fig. 5B is more monodisperse and the shoulder at 9.5 mL is less intense than the chromatogram shown in Fig. 5A. Fractions loaded in an SDS-PAGE indicate that protein bands were washed out (100 kDa, 75 kDa and 37 kDa bands Fig. 5B) compared to after Ni-NTA enrichment in Fig. 5A. Nevertheless, fraction from the shoulder at 9.5 mL (1 and 2, Fig. 5A) revealed a diffuse band between 150 kDa and 250 kDa, also present in Fig. 5B. The main band with an apparent molecular weight of 55 kDa, corresponding to hSGLT1 (ca. 75 kDa by mass spectrometry, Fig. S1E) appears after the first elution in Fig. 5B. In addition, to monitor our protein of interest we performed immunodetection of the hSGLT1-eGFP fusion protein (anti-eGFP yielding a band at apparent molecular weight of 75 kDa, Fig. S2), and the hSGLT1 protein (anti-hSGLT1 and anti-His-tag yielding apparent molecular weight bands of 55 kDa, Figs S3 and S4, respectively). The yield of 1 mg/L of purified monodisperse hSGLT1 protein was obtained after Ni-NTA, FLAG-tag and gel filtration purification and 1–2 mg/L were obtained after Ni-NTA and gel filtration.

### Secondary structure of Fos-12 solubilized hSGLT1

**Figure 6:**
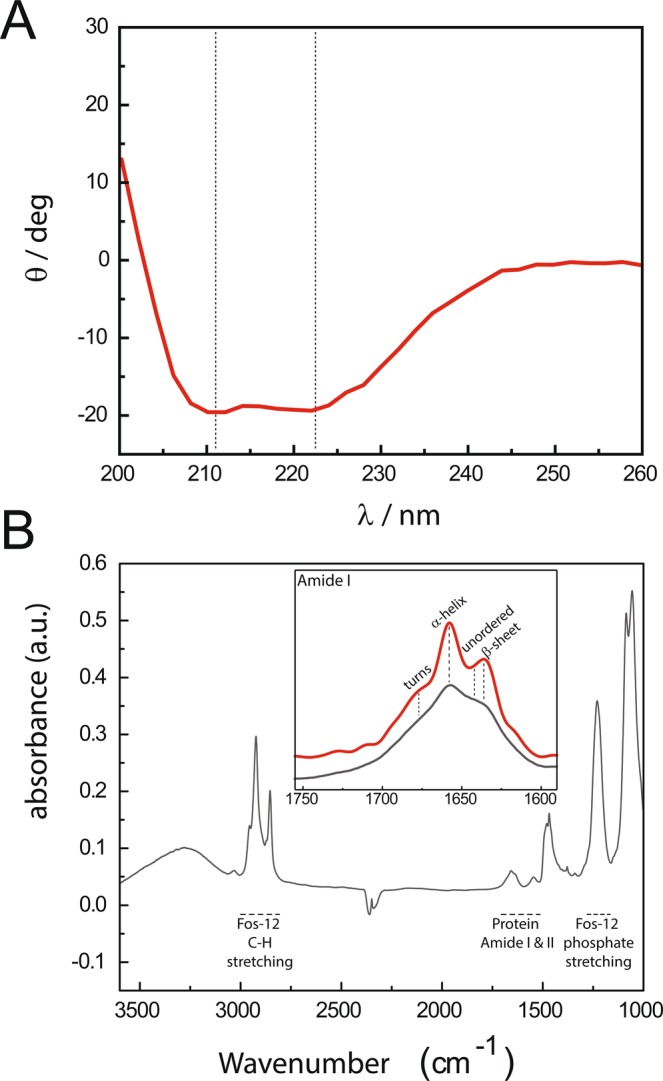
hSGLT1 secondary structure. (**A)** CD spectrum of WT hSGLT1 at 15 µM in 150 mM TKCL at pH 7.6. (**B**) FT-IR spectrum of purified WT hSGLT1 in detergent micelles. Complete FT-IR spectrum of WT hSGLT1 after drying it out under N_2_ stream. Amide I region is magnified and specific regions are represented: (α) α-helix at 1657 cm^−1^ and (β) β-sheet structure at 1635 cm^−1^. In black, dry FT-IR spectrum of WT hSGLT1 and, in red, dry FT-IR spectrum after deconvolution.

To assess the secondary structure of Fos-12 solubilized hSGLT1 (Fig. 6), we used circular dichroism (CD, Fig. 6A) and ATR-FTIR spectroscopies (Fig. 6B). CD spectrum for Fos-12 solubilized hSGLT1 (Fig. 6A) shows a characteristic α-helix structure indicated by the 210 and 222 nm minima. Secondary structure was estimated using K2D2 (**27**) and the calculated proportions secondary structures were: 85% α-helix, 14% β-sheet and 1% unordered, respectively. The 4000–1000 cm^−1^ ATR-FTIR spectrum provides detail on specific molecular details of the sample, such as amide I, II and Fos-12 (PO^−1^ and C-H) stretching (indicated in Fig. 6B). The amide I (1700–1600 cm^−1^) is widely used to assess protein secondary structure. In Fig. 6C we show the dry spectrum for Fos-12 solubilized hSGLT1 (grey line) and the band narrowing deconvolution (red line). Before deconvolution, a main peak is observed at 1650 cm^−1^ with a shoulder at 1630 cm^−1^. The 1650 cm^−1^ peak is characteristic of α-helix while the peak at around 1630 cm^−1^ is typical from β-sheet structure and, after deconvolution, those peaks are resolved successfully: α-helix peak is exactly at 1657 cm^−1^ while β-sheet peak is at 1635 cm^−1^. In summary, both methods show a predominantly α-helix secondary structure for Fos-12 solubilized hSGLT1, being higher in the sample in solution analyzed by CD compared to the dry sample by ATR-FTIR.

### Glucose transport by hSGLT1 in planar lipid membranes

**Figure 7:**
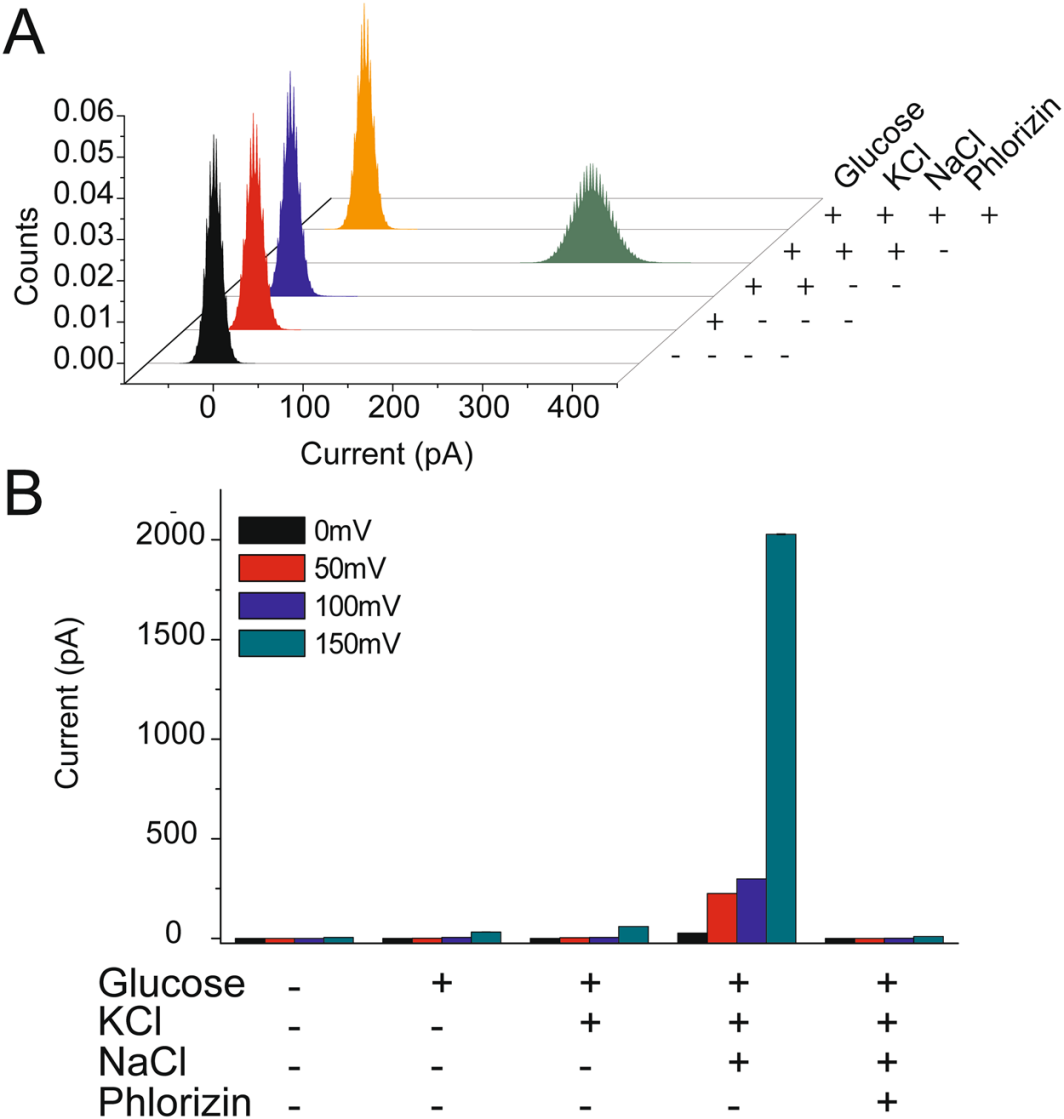
Functional characterization of hSGLT1 in planar lipid membranes. (**A**) Histogram of patch clamp measurements in planar lipid membranes at 100 mV. Histograms result from the evaluation of 4 seconds traces corresponding to: first addition of 10 ng of protein to the planar membrane (black); addition of 5 mM glucose to protein (red); addition of 200 mM KCl (blue); addition of 200 mM NaCl (green); addition of hSGLT1 inhibitor, phlorizin (100 µM, orange). All additions were done consecutively after protein was initially added (black histogram). (**B**) Overall data of planar lipid membranes measurements at 0 mV, 50 mV, 100 mV and 150 mV. An average of intensity was used after 4 seconds of recording.

The activity of Fos-12 solubilized and purified hSGLT1 needs to be tested. A transport assay was done in DPhPC planar lipid membranes (Fig. 7). Protein reconstitutes in a DPhPC lipid bilayer, ideally a single protein, and currents due to subsequent addition of hSGLT1 co-substrates and inhibitors were recorded (Fig. 7A). Histograms for currents values at +100 mV after the addition of 200 mM K^+^ are not significantly different (ca. 0 pA) from those with 200 mM K^+^ and 5 mM glucose as hSGLT1 co-substrate (Fig. 7A). Adding 200 mM Na^+^ as the second co-substrate increases the current values to ca. 300 pA. This large current change is unlikely to be caused by single protein insertion rather than the activity of multiple transporters inserted simultaneously in the DPhPC bilayer. Finally, the hSGLT1 activity was specifically inhibited to basal values (ca. 0 pA) using 100 µM phlorizin. The same trend was observed when the experiments were performed at +50, +100, and +150 mV (Fig. 7B and Supplementary Information).

## Discussion

Membrane proteins are challenging structural biology targets, especially human membrane proteins. Outstanding advances have been achieved in the structural biology field for secondary transporters, especially by resolving structures of prokaryote homologues as a first approximation, such as the lactose and melibiose permeases^19,20^. In the APC/SSS families, the structure of substrate-bound inward conformation for vSGLT has helped understanding the molecular alternating access mechanism of sodium-sugar transport^18^. The structure of vSGLT confirmed the prediction of 14 TM topology with both N- and C-termini facing the extracellular space^21^. The core structure for vSGLT is formed by two inverted repeats domain consisting of 5 TM helices each (TM2-6 and TM7-11) which is a very similar folding that has been observed for other non-active glucose transporters of 12 TM helices like in GLUT1,3,5^22^. vSGLT and hSGLT1 present a high sequence identity and similarity (32 and 75%, respectively), but structural biology efforts have not yielded the 3D structure for hSGLT1, although overexpression of this transporter has been achieved in *Pichia*^23^. At present, the lack of 3D structural data on hSGLT1 prevents the understanding of specific structural details on human sodium-glucose transport. A main trait of hSGLT1 still remains controversial, which is the large cytosolic loop between helices TM13 and TM14, which has even been hypothesized as extracellular loop key to the formation of a vestibule for the sugar substrates^24,25^. Structural biology of hSGLT1 is key for the development of effective pharmacological strategies directed to GGM, diabetes, and cholera, and other diseases dependent on intestinal glucose absorption^4,16,17^.

Here we describe a *P. pastoris* based strategy to produce large amounts of recombinant functional hSGLT1 for biochemical and biophysical studies, as a starting material for structural biology studies. Our *P. pastoris* strategy includes several approaches for a quick screening of conditions to ease the expression and purification, secondary structure characterization, and functional assessment of any membrane protein of choice using *P. pastoris* as expression host. Regarding clone selection, multiple copy integration events are more likely to occur (1–10% frequency) when the transformation method is electroporation and high amount of linearized vector is used (in the microgram range)^26^. Higher number of plasmid integration events are expected to yield higher protein expression^27^, arguing for the relevance of the clone screening step (Fig. 2). With our approach we have been able to select the best expressing *Pichia* strains for the non-fluorescent and fluorescent constructs (Fig. 2). Overexpression of membrane proteins easily leads to protein aggregation and accumulation in inclusion bodies or in specific degradation compartments (yeast vacuole)^13^. To monitor subcellular localization, imaging techniques taking advantage of the GFP fluorescence allowed us to detect non-aggregated eGFP-tagged proteins in membrane compartments different from the vacuole (Fig. 3). Previous studies on hSGLT1 expressed in *Pichia* identified Fos-12 as the most suitable detergent for solubilization^23^. Our detergent screening (Fig. 4) did not find a better suited detergent, but we provide insightful detail on the most adequate solubilization ratio (yeast membranes:detergent, see methods section for details). We have obtained a monodisperse hSGLT1 protein suitable for biophysical and biochemical studies (Fig. 5 and Supplementary Information), with a predominant α-helix secondary structure (Fig. 6) expected for a protein with a 14TM predicted topology.

Specifically for hSGLT1, and in general for membrane proteins, we have used several experimental setups for membrane protein expression, such as the use of yeast as an inexpensive expression host^13^, GFP-based strategies^28^, rapid clone selection strategies^29^ and accessible secondary structure characterization, such as CD. Additionally, we have included in the structural biology pipeline strategies such as quick secondary structure assessment of detergent solubilized membrane proteins using FTIR spectroscopy and mathematical deconvolution (Fig. 6B), which could be of further use in liposome reconstituted membrane proteins^30^. We have been able to assess the functionality of a detergent-solubilized membrane protein transporter without the hassle of liposome reconstitution, as a limiting step for protein function validation in structural studies^31,32^. Fluorescence and radioactivity experiments have been used to indirectly assess protein function using hSGLT1 purified from *Pichia* solubilized in detergent and reconstituted into liposomes^23,33^. Indirect assessments of substrate and/or inhibitor binding/uptake function using Trp are not devoid of controversy, mainly because the lack of specificity and the need liposome reconstitution of the protein to assess sugar uptake, and mutagenesis to validate the specificity of binding in the presence of substrate or inhibitors such as phlorizin^33^. Phlorizin is the inhibitor of choice for hSGLT1, but the characteristic aromatic nature for this compound overlaps with the protein intrinsic Trp emission range, preventing the usage of this method for quick validation of hSGLT1 in detergent-solubilized or liposome reconstituted systems^23,33^. Our approach to use lipid planar membranes has been widely used as an electrophysiology method for membrane active peptides^34^, ion channels^35^, or proteins with a high electrogenic potential^36^. In this study, we have used this method not as a single channel measurement, but as a quick electrophysiological assessment for function of a secondary active sugar co-transporter, such as hSGLT1. Although our lipid planar membranes setup is home-assembled, electrophysiological devices ready for pharmacological high-throughput screening of ion channels, and transporters are becoming readily available, an in fact some of them are being used to specifically assess hSGLT1 pharmacology^37^. We believe our screening strategy for protein expression and purification, including secondary structure and functional characterization with detergent solubilized protein will be of use for biophysics and structural biology laboratories requiring high amounts of membrane proteins, especially when considering human or mammalian orthologues. For hSGLT1, this strategy has produced unsuccessful crystallization attempts, which is the limiting step before tridimensionl high-resolution structure.

## Methods

### Materials

The *P. pastoris* strain (SMD1168H) was supplied by Thermo Fisher Scientific. Restriction enzymes *NdeI*, *Not1* and *PmeI* were from New England Biolabs (London, Great Britain). Plate reader was FLUOstar optima from BMG Labtech (Germany, Ortenberg). Superose 6 Increase 10/300 GL, Superdex 200 10/300 GL and Ni-NTA column were obtained from GE Healthcare. Phlorizin, Glucose and FLAG-tag resin were from Sigma-Aldrich. Detergents were purchased from Anatrace and kept at −20 °C. All other non-specific chemical reagents were of analytical grade and obtained from commercial source.

### Construction of expression vectors

The full-length hSGLT1 with a FLAG tag position at D_574_ was cloned into pP expression vector (derived from pPCIZB vector). The FLAG tag position changed the natural peptide sequence from D_574_ AEEN to D_574_ YKDDDDK. Coding DNA of hSGLT1 was amplified by polymerase chain reaction using the following primers: sense, 5′-ATT-AAG-CAT-ATG-GAC-AGT-AGC-ACC-TGG-3′, and antisense, 5′-TTA-ATA-TGC-GGC-CGC-GGC-AAA-ATA-TGC-ATG-GCA-AAA-3′. The cDNA sequence of hSGLT1 was cloned into NotI and NdeI sites of pP and pP-eGFP. The N248A mutant was done by KOD polymerase Quikchange reaction using pP-hSGLT and pP- as template and with the following primers: sense, 5′GTGTCTGATGGCGCCACCACCTTTCAGG3′, and antisense, 5′ CCTGAAAGGTGGTGGCGCCATCAGACAC 3′. The final expression products were hSGLT1 fused to 8xHistidine tag and hSGLT1 fused to an enhanced GFP (eGFP) followed by a 8XHis-tag. Both resulting plasmids were transformed into *E. coli* cells for further amplification and purification of the resulting plasmid. Both plasmids constructs were verified by DNA sequencing.

### Electroporation of *P. pastoris* with plasmid pP-hSGLT1 and pP-hSGLT1-eGFP

The purified DNA samples of pP-hSGLT1 and pP-hSGLT1-eGFP were linearized by *PmeI* digestion at 37 °C overnight. Linearized DNA was purified by enzyme removal column in order to remove remaining salts which hamper electroporation.

YPD medium (10 mL) was inoculated with *P. pastoris* strain SMD1168H and culture was grown overnight at 30 °C. The culture was transferred into 500 mL of fresh YPD at 30 °C until reaching an OD_600_ of 1. Cells were harvested by centrifugation at 2.000 g at 4 °C for 5 minutes and were suspended in YPD medium plus HEPES (pH 8.0, 200 mM) and 5 mM DTT in a new flask. The culture was incubated at 30 °C, 100 rpm for 15 minutes. 150 mL of cold sterile water was added into the culture and it was centrifuged at 2000g at 4 °C for 5 minutes. The cell pellet was suspended in 250 mL of cold sterile water and centrifuged. The pellet was suspended again in 20 mL of ice-cold sterile sorbitol 1 M. Finally, the culture was centrifuged and the resulting pellet was suspended in 1 mL of ice-cold sterile 1 M sorbitol. All suspensions must be done by gently shacking or slowly pipetting. Competent cells were kept in ice and used immediately or aliquoted and frozen at −80 °C.

For electroporation, the suspension of competent cells (40 µL) was mixed with lyophilized linearized DNA (24 µg) from *PmeI* digestion and transferred to an ice-cold electroporation cuvette. The cuvette with the mixture of cells and DNA was incubated on ice for 5 minutes. Cells were pulsed at 1.5 kV and 1 mL of ice-cold sorbitol was added immediately. Electroporated cells were transferred to recover in an incubator at 30 °C at 100 rpm for 3–4 hours. 200 µL of these cells were plated on YPD medium with 100 and 500 µg/mL of zeocin and incubated at 30 °C until colonies appear.

### Selection of multiple copy recombinant genes of P. pastoris transformants by serial dilution: zeocin viability

Transformed colonies of pP-hSGLT1 were streaked in a new YPD medium plate with 100 µg/mL of zeocin at 30 °C for 48 hours. Each transformant colony and a negative control (non-transformed SMD1168H colony) were inoculated in 5 mL of YPD medium and were left to grow at 30 °C overnight. Cells were harvested by centrifugation at 2,000 × g and resuspended in distilled-sterilized water. OD_600_ measures were taken for each culture and 1 mL was set aside to a final normalized OD_600_ of 1. Serial dilutions were done for each culture: 10^0^, 10^−1^, 10^−2^, 10^−3^, and 10^−4^. A drop test of 5 µl of each colony and their dilution was done in YPD medium plate with 100 and 500 µg/mL of zeocin. Plates were left to grow at 30 °C for 48 hours or until colonies appeared.

### Selection of multiple copy recombinant genes of *P. pastoris* transformants by in-plate induction

Transformed colonies of pP-hSGLT1-eGFP were streaked in a new YPD medium plate with 100 µg/mL zeocin at 30 °C for 48 hours. Each transformant colony, a positive control (pP-eGFP empty vector) and a non-transformed *P. pastoris* colony (SMD1168H) was inoculated in 5 mL of YPD medium and was left to grow at 30 °C overnight. Cells were harvested by centrifugation at 2000 × g to remove YPD medium and suspended in minimal methanol (MM) medium. OD_600_ measurements were taken for each culture and 100 µl were set aside and normalized to a final OD_600_ of 1. A drop test of 5 µl of each colony was done in MM plates which were transferred to an incubator at 30 °C for 48 hours. The plate was transilluminated with an eGFP fluorescence filter at 550 nm.

### Subcellular protein localization: Confocal microscopy

A selected expression clone of pP-hSGLT1-eGFP and a transformed pP-eGFP empty vector clone were inoculated in 25 mL of MM medium at 30 °C overnight. Cells were harvest by centrifugation at 2000× g and suspended in 25 mL of MM medium. Cultures were transferred back to an incubator at 24 °C for 48 hours. Small aliquots (50 µL) were removed at different times: 4, 8, 12 and 24 hours. Cells were normalized to and OD_600_ of 1. A drop of 5 µl of cells was put on a slide covered with a coverslip. A bright field picture and a fluorescence picture were taken at higher magnification after adding a drop of immersion oil as explained in^12^.

### Membranes preparation and large scale cell cultures

Large scale *P. pastoris* culture was grown in 200 mL of buffered minimal glycerol (BMG) medium at 24 °C for 24 hours. The culture was transferred into 4 L of BMG medium and was left to grow for 48 hours but adding Glycerol 1% (w/v) after 24 hours. The culture was spun down at 2,000 × g and was suspended into buffered minimal methanol (BMM) medium and transferred to an incubator at 24 °C for 20–24 hours for protein induction. The culture was harvested after centrifugation at 2,000 × g and suspended in distilled water to remove traces of BMM. Culture was spun down once more at 2,000 × g and the cell pellet was frozen with liquid nitrogen and stored at −80 °C.

The cell culture was slowly thawed at 4 °C. Up to 80 g of cells were resuspended with a vortex in a total volume of 175 mL of ice-cold breaking buffer (50 mM Tris-HCl, 1 mM EDTA, 5% (w/v) Glycerol, 0.1 mM PMSF, 10 mM DTT). Protease inhibitor cocktail (Complete, Roche) and an equal volume of glass beads (0.5 mm) were added to the suspension and cells were broken by mechanical disruption in a bead beater at 4 °C. Up to 20 cycles of 1 minute were carried out for cell disruption with 2 minutes interposition after each cycle to prevent heating. Unbroken cells were removed by centrifugation at 3,000 × g for 10 minutes. The supernatant was centrifuged at 100,000 × g for 1 hour to recover the membrane fraction, which was then flash frozen with liquid nitrogen.

### Detergent Screening

Membranes were thawed slowly and homogenized in TKCL (20 mM Tris-HCl, 150 mM KCl, 1 mM DTT pH 7.6) and solubilized for 2 hours at 4 °C by adding specific detergents at 3x critical micellar concentration (CMC) for initial screening (detergents are listed in Fig. 4a). Non-soluble aggregates were removed by centrifugation at 25,000 × g for 30 minutes and up to 200 µL of each sample were taken to a plate reader and a fluorescence measure was recorded with an excitation/emission filter set at 485 nm and 520 nm respectively. For each tested detergent, two measures were recorded: total solubilized membranes before ultracentrifugation and supernatant of ultracentrifugation so a percentage of fluorescence recovery could be calculated. A further analysis was done by fluorescence size exclusion chromatography (FSEC) for most commonly used detergents as described in^13^, such as: Fos-Choline-12 (Fos-12), n-Dodecyl-β-D-Maltopyranoside (DDM), n-Decyl-β-D-Maltopyranoside (DM), n-Octyl Glucoside (OG) and N,N-Dimethyldodecylamine N-oxide (LDAO)at 1% (w/v) concentration. Membranes were homogenized and solubilized with the same conditions stated before. Non-soluble aggregates were removed by centrifugation at 50,000 × g for 30 minutes. 5 µL of the resulting supernatant were injected into a Superose 6 column through a HPLC system. A high sensitivity program was used with an excitation/emission wavelength at 480/510 nm.

### Protein purification

Membranes were thawed slowly and homogenized in TKCL buffer at a specific ratio (w/v) of 0.16 (grams of membrane/total volume of solubilization) at 1.2% (w/v) of Fos-12 for 2 hours. Solubilized membranes were spun down at 50.000 × g for 45 minutes and the clean supernatant was recovered. The supernatant was poured to a 5-mL bed (1-cm diameter) of Ni^+2^ chelate to resin (nickel-nitrilotriacetic, Ni-NTA) which was previously equilibrated with 5 column volumes (CV) of TKCL at 1.2% of Fos-12 (w/v). The resin with the solubilized membranes was left in batch mode for 3–4 hours at 4 °C with slow shaking in a beaker. The resin was transferred back into a column and left to pack again by starting the flow. A washing step of 5 CV of TKCL at 0.2% (w/v) of Fos-12 without imidazole was done followed by another washing step of 10 CV but with 10 mM imidazole. The protein was eluted with an elution step of 5 CV of TKCL at 0.2% (w/v) of Fos-12 with 150 mM imidazole (higher imidazole concentrations can be used up to 250 mM). His-tagged eluted protein fractions were pooled and concentrated with a Centricon (Millipore) to 0.5–1 mL final volume. Concentrated fraction was centrifuged at 50.000 × g for 20 minutes to pellet any aggregates and then applied to a Superdex 200 HR 10/30 gel filtration column equilibrated previously in TKCL at 0.2% Fos-12 (w/v). An ÄKTA purifier system was used for the SEC purification at a flux of 0.4 ml/min. Eluted fractions of the elution were pooled together and stored at −4 °C.

Another affinity purification step (FLAG-tag) may be added between the Ni-NTA purification and the SEC depending on the required sample purity. Initially, imidazole was lowered down from the purified protein sample from the Ni-NTA with a Centricon to 10 mM. Ni-NTA purified sample was poured to 4-mL bed (1.5-cm diameter) of FLAG-tag resin (Sigma) which was previously equilibrated in 2 CV of high salt TKCL (20 mM Tris, 500 mM KCl, 1 mM DTT, pH 7.6) at 0.2% (w/v) of Fos-12 and left in batch mode overnight at 4 °C. The resin was repacked by restarting the flow and washed with high salt TKCL until no protein was observed in the washes. Elution was done with 5 CV of FLAG-tag elution buffer (200 mM Glicine, 150 mM KCl, 1 mM DTT pH 3.5) at 0.2% (w/v) of Fos-12 which was immediately neutralized with 1 M Tris-HCl at pH 8.0 to equilibrate the final pH.

### SDS-PAGE and Immunoblotting

8 or 10% SDS-PAGE gels were run at 120 V. For in-gel fluorescence of eGFP fusion proteins gels were illuminated using blue light transilluminator. Subsequently gels were commassie blue-stained or transferred using a semi-dry blot device to nitrocellulose membranes. Nitrocellulose membranes were blocked with 5% BSA for 1 hour. Primary antibodies against hSGLT1 (1:5000 hSGLT1-rabbit, Cat. No. AP00343PU-N Acris antibodies), eGFP (1:1000, DHSB Cat. No. GFP-G1), and His-tag (1:8000, DHSB Cat. No. P5A11). Secondary antibody incubation was followed up by detection using the Immobilon Western kit (Millipore).

### Structural characterization: Fourier transform infrared spectroscopy (FTIR) spectroscopy and circular dichroism (CD)

Purified protein in micelles in TKCL at 3 mg/mL was used to record FT-IR and CD spectra. CD spectrum of WT hSGLT1 was taken at 15 µM in 150 mM TKCL at pH 7.6. Measures were taken in cuvette with an optical path of 1 mm in a JASCO J-715 spectropolarimeter. FTIR spectra were recorded with a Varian 7000Fourier transform spectrometer equipped with a attenuated total reflection (ATR) diamond crystal at room temperature at a resolution of 2 cm^−1^. The sample chamber was purged continuously with N_2_ in order to remove water vapor.

### Planar lipid membranes preparation and transport studies

Planar bilayers were formed by apposition of two monolayers prepared from a solution of 1% pure diphytanoyl phosphatidylcholine (DPhPC) in pentane. 10 µL lipids at 5 mg/mL were added on 70–90 µm diameter orifice in the 15 µm thick Teflon partition that separated two identical chambers (**25, 26**). The orifice was pretreated with 1% hexadecane in pentane before adding the lipids. Aqueous solutions of chambers were buffered with 50 mM HEPES at pH 7 and 100 mM KCl. All measurements were done at room temperature (23–25 °C). Control experiments assured that lipid bilayers without any protein addition were totally impermeable. Membrane permeabilization was achieved by adding 1–5 µL of purified protein (hSGLT1) at 2.5 mg/mL in TKCL (20 mM Tris, 150 mM KCl, 1 mM DTT pH 7.6) at 0.2% Fos-12 into the chamber.

An electric potential was applied using Ag/AgCl electrodes in 2 M KCl, 1.5% agarose bridges assembled within standard 250 µL pipette tips. The potential was defined as positive when it was higher on the side of the protein addition (*cis* side), whereas the *trans* side was set to ground. An Axopatch 200B amplifier in the voltage-clamp mode was used to measure the current and the applied potential. The chamber and the head stage were isolated from external noise sources with a double metal screen. Current traces were recorded at several applied voltages: 150, 100, and 50 mV. Small intensities (5–20 pA) revealed protein insertion into the membrane and, in that case, substrates (glucose and sodium) and inhibitor (phlorizin) were added successively. In particular, 200 mM NaCl (specific-transport ion) was used and intensity was recorded at each specified voltage. Same protocol was used for 200 mM KCl (non-specific-transport ion), 5 mM glucose, and 100 µM phlorizin.

### Mass spectrometry

WT hSGLT1 purified protein after FLAG-tag and SEC chromatography was treated with DTT 500 mM for 15 minutes at room temperature. Afterwards, protein was precipitated with chloroform and methanol and then dissolved in 20 μL 90% formic acid for immediate analysis by size-exclusion liquid chromatography with positive ion electrospray ionization mass spectrometry (LC-MS)^38^. The column (4.6 × 300 mm; Tosoh SW2000) was equilibrated in chloroform/methanol/1% aqueous formic acid (4/4/1; v/v) at 40 °C with a flow rate of 250 μL/min. and eluted into the source of a linear ion-trap mass spectrometer (LTQ, Thermofisher Scientific). Protein mass spectra were deconvoluted using BioMultiview software (Sciex).

## Supplementary information

Supplementary Information
