# Supplementary material for: Structural biology workflow for the expression and characterization of functional human sodium glucose transporter type 1 in Pichia pastoris
Source: Sci Rep. 2019 Feb 4;9:1203. doi: 10.1038/s41598-018-37445-2 (PMC6362292; doi:10.1038/s41598-018-37445-2)
Supplement: Supplementary file 1 — Supplementary Information [file 41598_2018_37445_MOESM1_ESM.pdf]

Structural biology workflow for the expression and characterization of functional human sodium glucose transporter type 1 in *Pichia pastoris*.

Albert Suades[1,+], Antonio Alcaraz[2], Esteban Cruz[1], Elena Álvarez-Marimon[1], Julian P. Whitelegge[3], Joan Manyosa[1], Josep Cladera[1], Alex Perálvarez-Marín[1,*]

[1] Biophysics Unit, Department of Biochemistry and Molecular Biology, School of Medicine, Universitat Autònoma de Barcelona, 08193 Cerdanyola del Vallés, Catalonia, Spain

[2] Laboratory of Molecular Biophysics, Department of Physics, Universitat Jaume I, 12071 Castellón, Spain

[3] The Pasarow Mass Spectrometry Laboratory, The NPI-Semel Institute, David Geffen School of Medicine, UCLA, 760 Westwood Plaza, Los Angeles, CA 90095, USA

[*]**Correspondence to:** Alex Perálvarez-Marín, Ph.D. **email:** alex.peralvarez@uab.cat **Address:** Unitat de Biofísica. Facultat de Medicina. Universitat Autònoma de Barcelona. 08193 Cerdanyola del Vallés. Catalonia. Spain. **ORCID:** 0000-0002-3457-0875

[+]**Current Address:** Department of Biochemistry and Biophysics, Stockholm University, SE-10691, Stockholm, Sweden

Supporting Information

Figure S1. Purification Overview. A. Representative Ni-NTA purification of WT-hSGLT1. Ladder (1); Flow through (2); Washes without imidazole (3-4); Washes at 10 mM Imidazole (5-6); Elution with 150 mM Imidazole lanes (7-11); Elution with 250 mM Imidazole (12-13). All elution lanes represents fractions of 1mL elution except (12-13) which were done at 10 mL. **B**. SEC repurification of WT-hSGLT1 (red) and N248A (black) with its corresponding fractions (1 to 6) in SDS-PAGE after Ni-NTA purification. **C.** Superdex 200 HR calibration using gel filtration standards, such as blue dextran, apoferritin, alcohol deshydrogenase, albumin, and carbonic anhydrase (grey solid chromatogram). hSGLT1 purification is indicated in orange, and the main peaks are represented as arrows. **D.** Molecular weight estimation of the two main peaks for hSGLT1 using the calibration standards as reference **E.** MALDI-TOF mass spectrometry of the excised band for WT-hSGLT1 at 55 kDa apparent molecular weight in SDS-PAGE.

Figure S2. hSGLT1-eGFP immunodetection. Western Blot using anti-eGFP on 10% SDS-PAGE for Ni-NTA purification; 1: Starting material before centrifugation; 2: solubilized material; 3: Non-solubilized material; 4: Flow-through; 5: Wash at 10 mM Imidazole; 6-13: Elutions of Ni-NTA at 250 mM Imidazole; 14: Purified free eGFP.

Figure S3. hSGLT1 immunodetection. Western Blot using anti-hSGLT1 on 10% SDS-PAGE for Ni-NTA purification; Flow through (FT); Washes at 10 mM Imidazole (W1), Washes at 20 mM Imidazole (W2 and W3) ; Ni-NTA elutions at 250 mM Imidazole (E1-E3).

Figure S4. His-tagged protein immunodetection. Western Blot using anti-His-Tag on 8% SDS-PAGE for SEC fractions; 1-4; Elutions of SEC chromatography represented on Figure 5.

Figure S5. **Electrophysiology measurements of hSGLT1 in planar lipid membranes.** **A**. Traces at 50 mV. **B**. Traces at 100 mV. **C**. Traces at 150 mV. See figure inset for color details. **D**. Histogram of patch clamp measurements in planar lipid membranes at 50 mV. Histograms result from the evaluation of 4 seconds traces corresponding to: first addition of 10 ng of protein to the planar membrane (top); addition of 200 mM NaCl; addition of 200 mM KCl; addition of 5 mM glucose; addition of hSGLT1 inhibitor, phlorizin (100 µM, bottom). All additions were done sequentially after protein was initially added (top histogram).

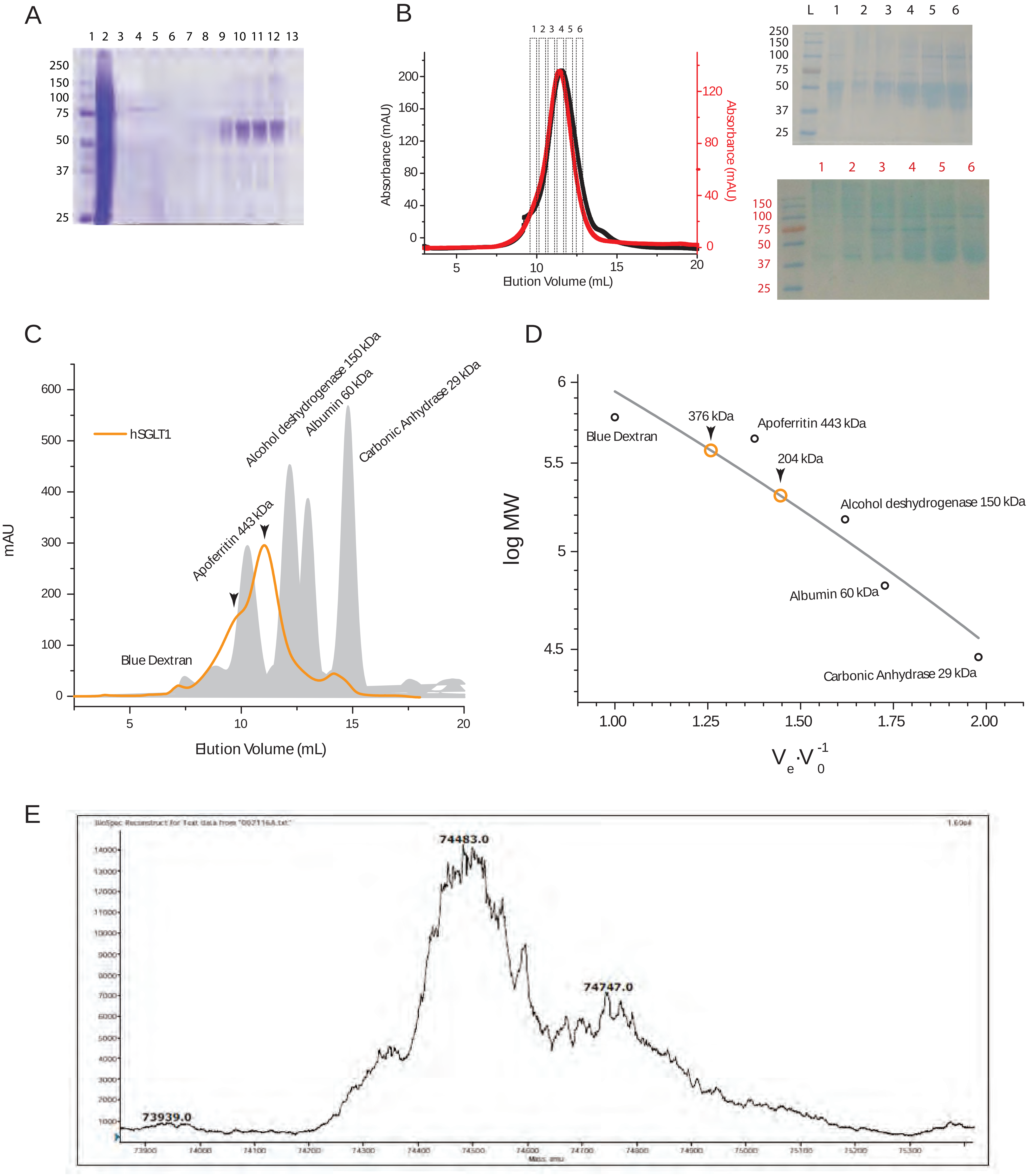
A
B
C
D
E
Absorbance (mAU)
Absorbance (mAU)
Elution Volume (mL)
hSGLT1
Blue Dextran
Apoferritin 443 kDa
Alcohol deshydrogenase 150 kDa
Albumin 60 kDa
Carbonic Anhydrase 29 kDa
mAU
log MW
376 kDa
204 kDa
74483.0
74747.0
73939.0

FIGURE S1

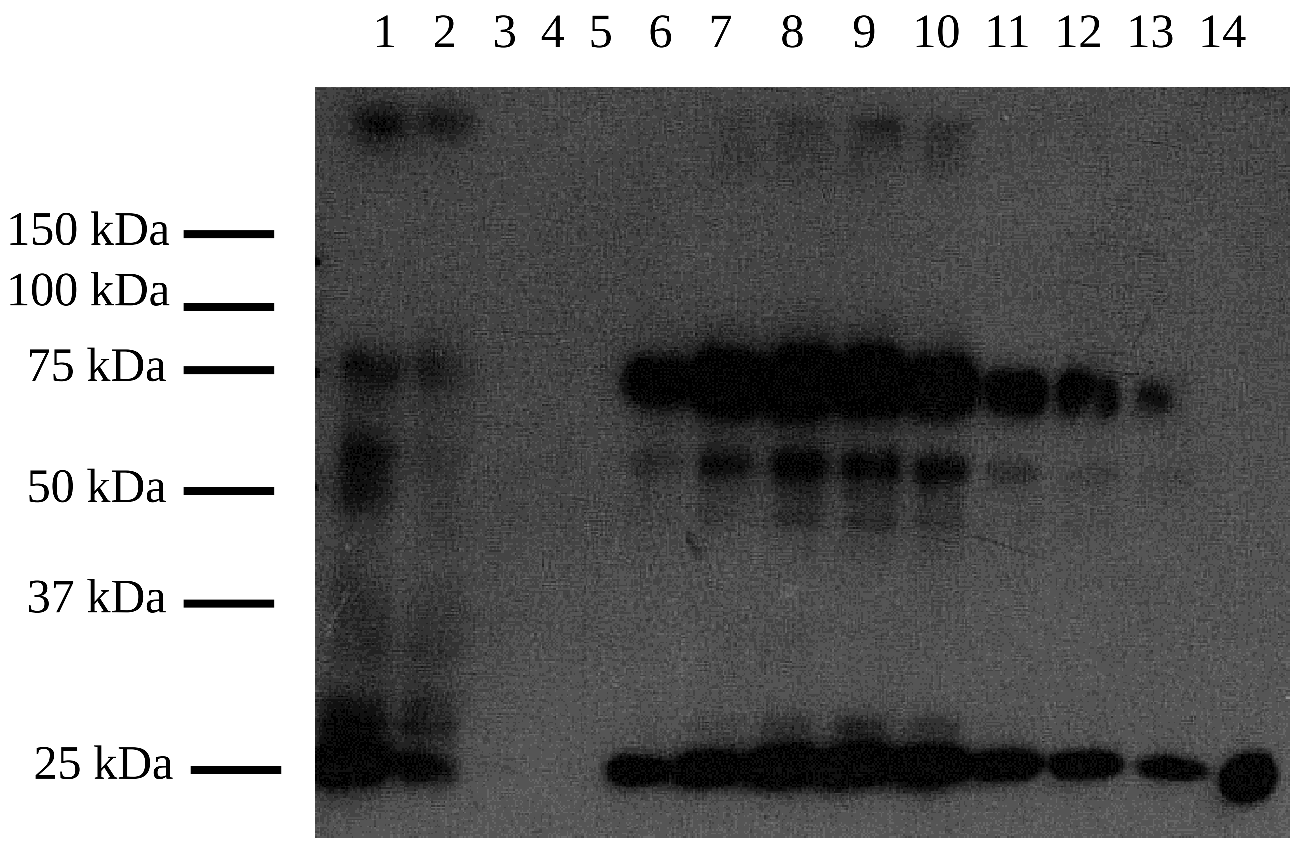
1 2 3 4 5 6 7 8 9 10 11 12 13 14
150 kDa
100 kDa
75 kDa
50 kDa
37 kDa
25 kDa

FIGURE S2

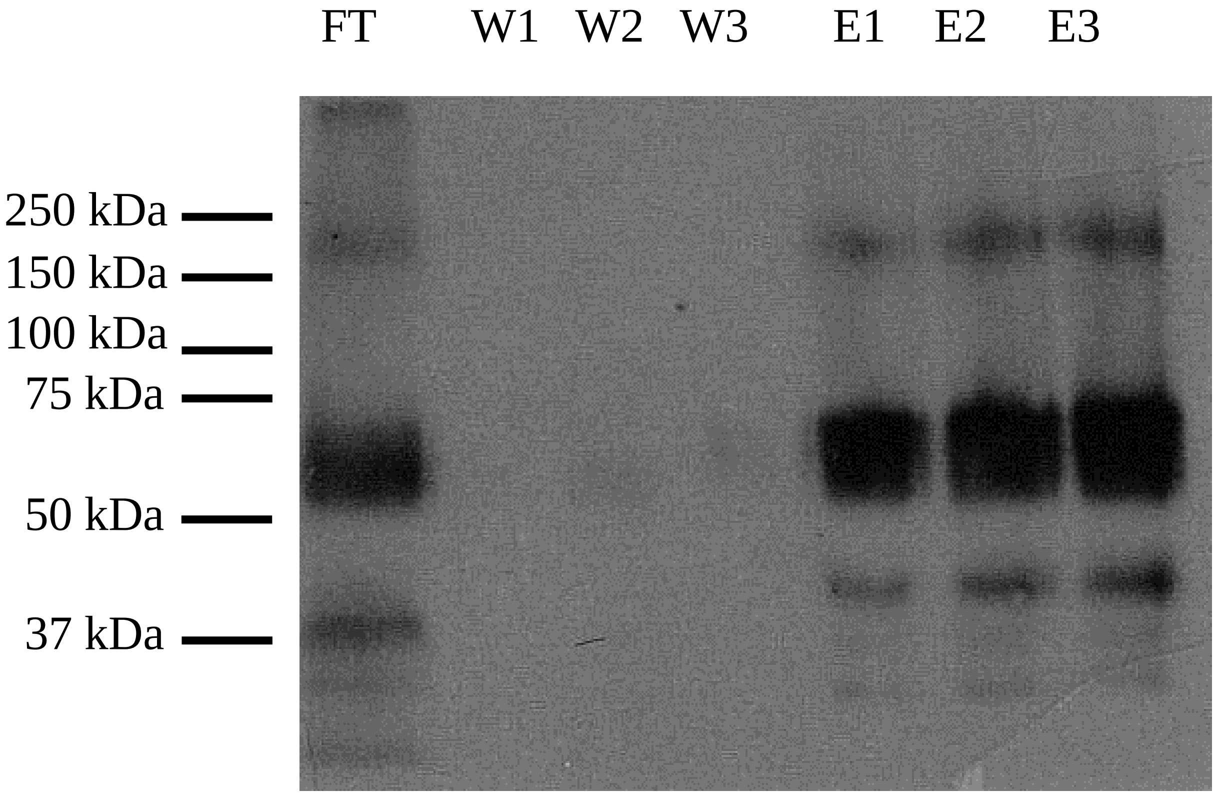

FIGURE S3

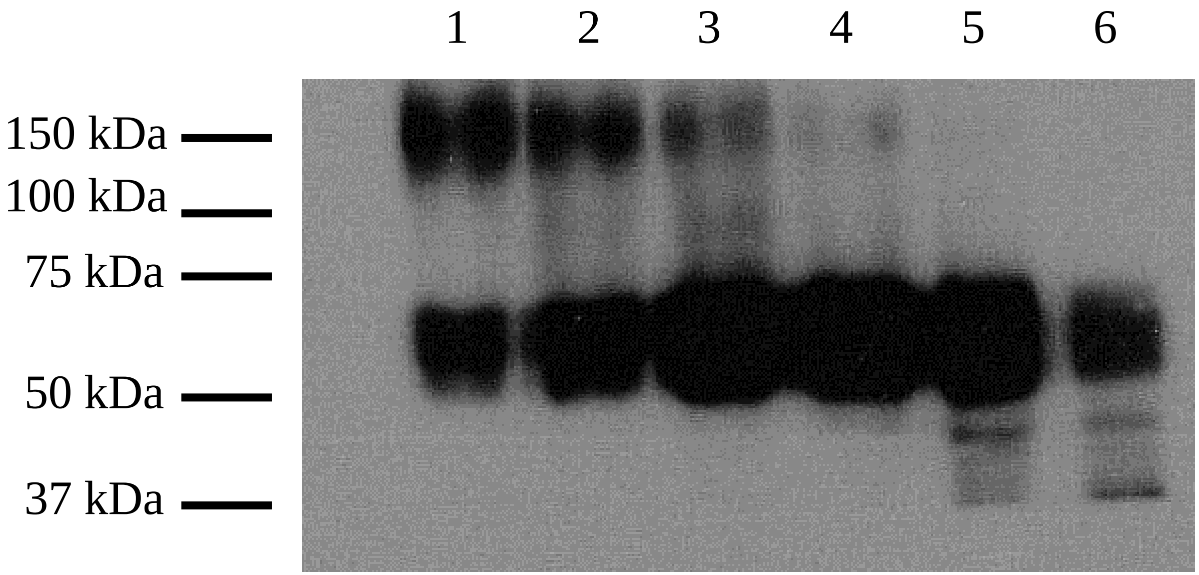

FIGURE S4

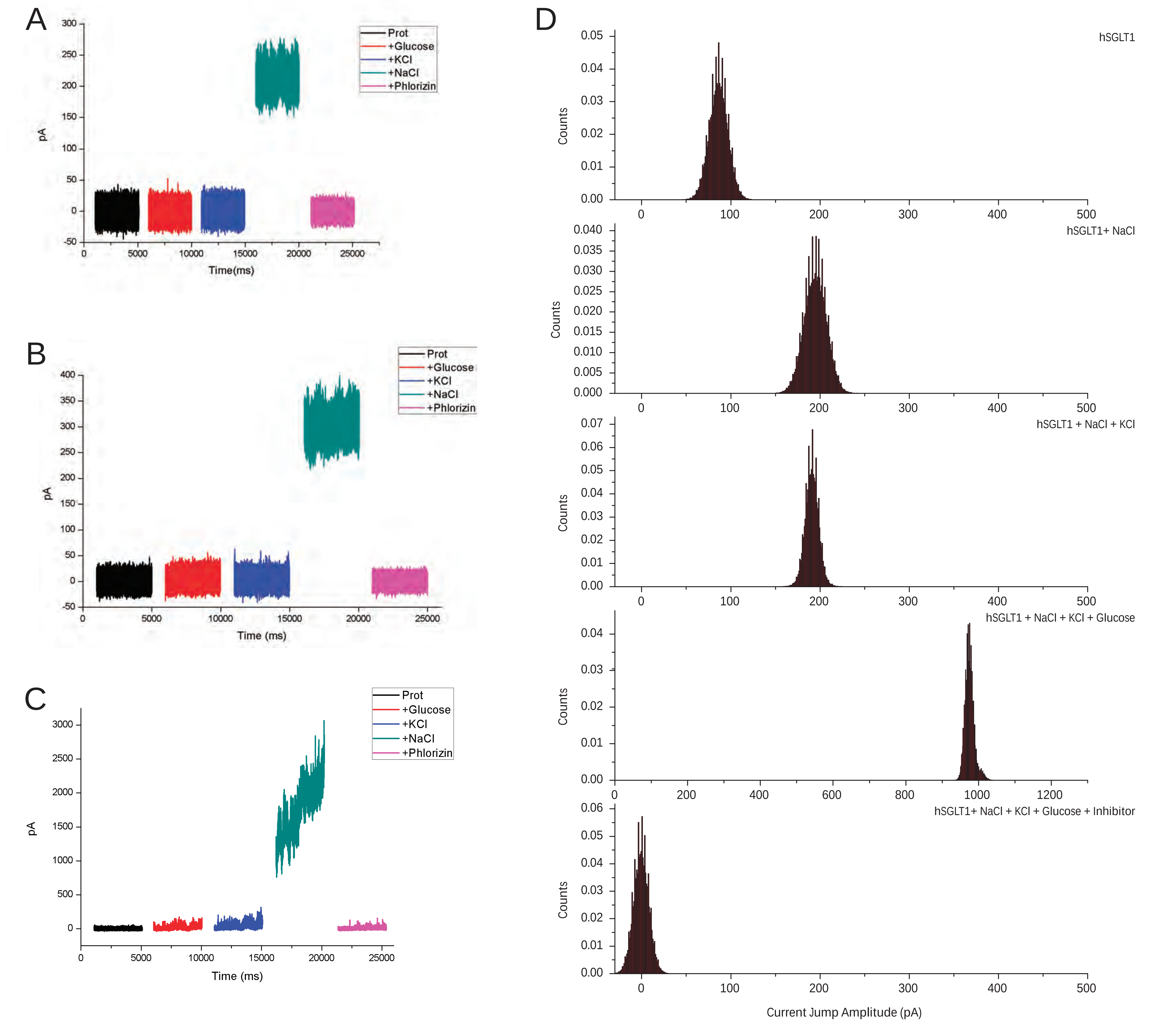
A
Prot
+Glucose
+KCl
+NaCl
+Phlorizin
pA
Time(ms)
B
Prot
+Glucose
+KCl
+NaCl
+Phlorizin
pA
Time (ms)
C
Prot
+Glucose
+KCl
+NaCl
+Phlorizin
pA
Time (ms)
D
hSGLT1
hSGLT1+ NaCl
hSGLT1 + NaCl + KCl
hSGLT1 + NaCl + KCl + Glucose
hSGLT1+ NaCl + KCl + Glucose + Inhibitor
Counts
Current Jump Amplitude (pA)

FIGURE S5
